# Supplementary material for: The genome sequence of a rove beetle, Othius punctulatus (Goeze, 1777)
Source: Wellcome Open Res. 2023 Nov 13;8:519. [Version 1] doi: 10.12688/wellcomeopenres.20338.1 (PMC11109532; doi:10.12688/wellcomeopenres.20338.1)
Supplement: Peer review history

# Reviewer report: Reviewer response for version 1

**DOI:** 10.21956/wellcomeopenres.22521.r76021

**Published:** 2024-05-20

**Authors:** Liang Lü

**Affiliations:** 1 Hebei Normal University, Shijiazhuang, China

I am happy to see one more rove beetle species with a high-quality genome. Actually, the author presents a simple article with a standard and widely accepted methodology, but not simple results, which were actually described in a very simple form. I recommend the editorial office consider its acceptance with minor or no revision. My only worry is about the identity of ths sample specimen, because the manuscript shows only in a lateral view, which does not expose many definite traits of Othius punctulatus. The author should show in dorsal view if it is still possible.

Are sufficient details of methods and materials provided to allow replication by others?

Yes

Is the rationale for creating the dataset(s) clearly described?

Yes

Are the datasets clearly presented in a useable and accessible format?

Yes

Are the protocols appropriate and is the work technically sound?

Yes

Reviewer Expertise:

rove beetle taxonomy and evolution

I confirm that I have read this submission and believe that I have an appropriate level of expertise to confirm that it is of an acceptable scientific standard.

# Reviewer report: Reviewer response for version 1

**DOI:** 10.21956/wellcomeopenres.22521.r80944

**Published:** 2024-05-15

**Authors:** Robert M Waterhouse

**Affiliations:** 1 University of Lausanne, and the Swiss Institute of Bioinformatics, Lausanne, Switzerland

This Data Note for the rove beetle presents a clear and comprehensive description of all the steps taken to generate the *Othius punctulatus *genome assembly spanning 870 Mb with 97% assigned to 10 chromosomal-level scaffolds including the X sex chromosome. The rationale behind the production of these reference data resources is clearly described, explaining the importance of genomic data and their contribution to improving our understanding of the biology, ecology, and evolution of *O. punctulatus* and other rove beetles. The results also include the mitochondrial genome assembly, I had not realized that it was now common to include the mitochondrial genome as a scaffold within the multifasta of a nuclear genome submission. The described data collection and analysis methods follow the best practices in the field and have delivered a high-quality complete and accurate chromosome-level reference genome. The manuscript methods provide detailed descriptions of the protocols employed for sample acquisition, DNA extractions, and sequencing of long reads and Hi-C data generation. The genome assembly is of one haplotype, with contigs corresponding to the second haplotype also deposited. The results are clearly presented along with the standard quality control analyses and summary statistics to be able to easily verify the high quality of the generated reference resources, which are freely accessible along with appropriate metadata in community open access genomic data repositories (the ENA and INSDC global mirrors).

Are sufficient details of methods and materials provided to allow replication by others?

Yes

Is the rationale for creating the dataset(s) clearly described?

Yes

Are the datasets clearly presented in a useable and accessible format?

Yes

Are the protocols appropriate and is the work technically sound?

Yes

Reviewer Expertise:

Arthropod genomics

I confirm that I have read this submission and believe that I have an appropriate level of expertise to confirm that it is of an acceptable scientific standard.

# Reviewer report: Reviewer response for version 1

**DOI:** 10.21956/wellcomeopenres.22521.r70462

**Published:** 2024-05-11

**Authors:** Yi-Ming Weng

**Affiliations:** 1 Department of Entomology, University of Wisconsin Madison, Madison, WI, USA

The genome report entitled "The genome sequence of a rove beetle, *Othius punctulatus *(Goeze, 1777)" is reviewed. This report includes brief and clear introduction about the species, followed by the result of genome assembly work. Despite of the high completeness according to BUSCO and Merqury, more (470) scaffolds were in the final assembly than I expected. Considering that the longest 10 scaffolds have accounted for the 10 chromosomes, I wonder if it is possible with more sequences to improve the assembly in the future to assemble the remaining scaffolds into the existing chromosomes.

Are sufficient details of methods and materials provided to allow replication by others?

Yes

Is the rationale for creating the dataset(s) clearly described?

Yes

Are the datasets clearly presented in a useable and accessible format?

Yes

Are the protocols appropriate and is the work technically sound?

Yes

Reviewer Expertise:

entomology, population genetics, evolutionary genomics

I confirm that I have read this submission and believe that I have an appropriate level of expertise to confirm that it is of an acceptable scientific standard.

# Reviewer report: Reviewer response for version 1

**DOI:** 10.21956/wellcomeopenres.22521.r80938

**Published:** 2024-05-07

**Authors:** Steven M Van Belleghem

**Affiliations:** 1 University of Puerto Rico, KU Leuven, Puerto Rico, Brazil

The paper presents a genome assembly for the rove beetle Othius punctulatus ith very high quality:

- **Total Assembly Length**: 870.5 megabases
- **Number of Scaffolds**: 470
- **Scaffold N50**: 97.1 megabases
- **Number of Contigs**: 669
- **Contig N50**: 7.7 megabases
- **Quality Value (QV)**: 59.6, indicating a high-quality assembly
- **k-mer Completeness**: 100%, suggesting excellent coverage of the genome
- **BUSCO Completeness**: 98.8% (with 97.3% single copy and 1.5% duplicated), using the endopterygota_odb10 BUSCO set, indicating that nearly all expected gene content is present
- **Mitochondrial Genome**: Also assembled and included within the genome data
- **Chromosomal Assignment**: 96.91% of the assembly sequence is assigned to 10 chromosomal-level scaffolds, representing 9 autosomes and the X sex chromosome.

The authors outline the significance of the dataset in enhancing our understanding of the beetle's evolutionary biology, ecological adaptations, and biodiversity.

 The protocols used for sample acquisition, nucleic acid extraction, sequencing, and genome assembly are thoroughly outlined and appropriate for the study. The methods incorporate advanced techniques like PacBio single-molecule HiFi long reads and Hi-C data, ensuring a robust and reliable genomic assembly. The work is technically sound, employing current best practices in genomic research.

 The paper provides detailed descriptions of the methods and materials used, including specific protocols for each stage of sample processing and data generation

 The datasets are clearly presented and accessible in a format that is usable for further analysis by the scientific community. The authors provide comprehensive assembly statistics, quality metrics, and chromosomal-level scaffolds.

Are sufficient details of methods and materials provided to allow replication by others?

Yes

Is the rationale for creating the dataset(s) clearly described?

Yes

Are the datasets clearly presented in a useable and accessible format?

Yes

Are the protocols appropriate and is the work technically sound?

Yes

Reviewer Expertise:

Genomics

I confirm that I have read this submission and believe that I have an appropriate level of expertise to confirm that it is of an acceptable scientific standard.

# Reviewer report: Reviewer response for version 1

**DOI:** 10.21956/wellcomeopenres.22521.r76025

**Published:** 2024-03-18

**Authors:** Luciano Palmieri

**Affiliations:** 1 Faculty of Agricultural Environmental and Food Sciences, Free University of Bolzen-Bolzano, Bolzano, Bolzano, 39100, Italy; 2 Department of Integrative Biology, University of Wisconsin-Madison, Madison, Wisconsin, USA

The text outlines the creation of a chromosomally complete genome sequence for *Othius punctulatus*, a species of rove beetle belonging to the Staphylinidae family. The rationale for creating the dataset is clearly described, emphasizing the importance of understanding the genomic makeup of *O. punctulatus* for advancing research in evolutionary biology, ecology, and biodiversity. The manuscript describes the species' distribution, habitat preferences, and behavioral patterns, underscoring the relevance of genomic data in unraveling the genetic basis of its ecological adaptations and evolutionary history.

 The protocols employed in sample acquisition, nucleic acid extraction, and sequencing are appropriate. The text provides detailed descriptions of the methodologies used, including sample preparation, DNA extraction, library construction, and sequencing. The integration of PacBio single-molecule HiFi long reads and Hi-C data for genome assembly and scaffold correction reflects a robust approach to genomic analysis. The manuscript also highlights the availability of publicly accessible protocols, ensuring transparency and reproducibility in experimental procedures. Sufficient details of methods and materials are provided, facilitating replication by other researchers. References to publicly available protocols and software tools enhance the comprehensibility and replicability of the experimental procedures outlined in the study.

 The datasets are provided in a usable and accessible format, enabling further analysis and exploration by the scientific community. Assembly statistics, quality metrics, and chromosomal-level scaffolds are clearly presented, providing insights into the genomic composition of *O. punctulatus*. The inclusion of metadata, barcode results, sequencing runs, and assembly statistics enhances the transparency and accessibility of the genomic dataset. The authors' meticulous approach to experimental design, execution, and data dissemination contributes to the advancement of knowledge in entomology and genomic biology.

Are sufficient details of methods and materials provided to allow replication by others?

Yes

Is the rationale for creating the dataset(s) clearly described?

Yes

Are the datasets clearly presented in a useable and accessible format?

Yes

Are the protocols appropriate and is the work technically sound?

Yes

Reviewer Expertise:

Coleoptera, Systematics, Biogeography, Biodiversity, Taxonomy, Entomology, Systematic Entomology, Insect Taxonomy, Evolution, Natural History, Molecular Phylogenetics, Invertebrate Zoology, Parasitoids, Macroevolution, Genome Assembly, and Phylogenomics.

I confirm that I have read this submission and believe that I have an appropriate level of expertise to confirm that it is of an acceptable scientific standard.
